# 

**Published:** 1808-02

**Authors:** 


### NEW MEDICAL PUBLICATIONS.

A Practical Treatise on Strictures and Diseases of the Prostrate Gland, &c. &c. By T. M. Caton. 2s.

Remarks on the Reform of the Pharmaceutical Nomenclature, and particularly on that adopted by the Edinburgh College. 2s.

